# Impact of macro-fiscal determinants on health financing: empirical evidence from low-and middle-income countries

**DOI:** 10.1186/s41256-019-0112-4

**Published:** 2019-08-09

**Authors:** Deepak Kumar Behera, Umakant Dash

**Affiliations:** 1 Indian Institute of Public Health, Public Health Foundation of India, 751013 Bhubaneswar, India; 2 Department of Humanities and Social Sciences, Indian Institute of Technology Madras, 600036 Chennai, India

**Keywords:** Health financing, Universal health coverage, Macro-fiscal policies, Global financial crisis, Revenue mobilization, Generalized method of moment

## Abstract

**Background:**

Health financing is a major challenge in low-and middle-income counties (LMICs) for achieving Universal Health Coverage (UHC). Past studies have argued that the budgetary allocation on health financing depends on macro-fiscal policies of an economy such as sustained economic growth and higher revenue mobilization. While the global financial crisis of late 2008 observed a shortage of financial resources in richer countries and adversely affected the health sector. Therefore, this study has examined the impact of macro-fiscal policies on health financing by adopting socioeconomic factors in 85 LMICs for the period 2000 to 2013.

**Methods:**

The study has employed the panel System Generalized Method of Moment model that captures the endogeneity problem in the regression estimation by adopting appropriate instrumental variables.

**Results:**

The elasticity of public health expenditure (PHE) with respect to macro-fiscal factors varies across LMICs. Tax revenue shows a positive and statistically significant relationship with PHE in full sample, pre-global financial crisis, middle-income, and coefficient value varies from 0.040 to 0.141%. Fiscal deficit and debt services payment shows a negative effect on PHE in full sample, as well as sub-samples and coefficient value, varies from 0.001 to 0.032%. Aging and per capita income show an expected positive relationship with PHE in LIMI countries.

**Conclusions:**

Favorable macro-fiscal policies would necessarily raise finance for the health sector development but the prioritization of health budget allocation during the crisis period depends on the nature of tax revenue mobilization and demand for health services. Therefore, the generation of health-specific revenues and effective usage of health budget would probably accelerate the progress towards the achievement of UHC.

**Electronic supplementary material:**

The online version of this article (10.1186/s41256-019-0112-4) contains supplementary material, which is available to authorized users.

## Background

Health financing^1^ is a major challenge in low-and middle-income countries (LMICs) for achieving Universal Health Coverage (UHC)^2^ [1]. Because LMICs allocate very less financial resources towards the development of the health sector which leads to higher catastrophic Out-of-Pocket (OOP) health expenditure and poor health services [2]. Additional file 2: Table S2 shows the trends of health financing indicators^3^ in LMICs and their level of achievement in terms of threshold target of health expenditure as suggested by Mathuare and Carrin [3]. In the year 2014–15, nearly 75% of LMICs Public Health Expenditure (PHE) is below 5% of Gross Domestic Product (GDP); while nearly 63% of middle-income countries’ PHE is below 15% of General Government Expenditure (GGE). Further, the average per capita PHE is 14.8 US$ in low-income, 81.7 US$ in low-middle income and 333.8 US$ in upper-middle income countries. The LMICs have achieved 86 US$ per capita PHE but nearly about 70% of countries are below the average of per capita PHE. In terms of PHE (as % of Total Health Expenditure), around 30% of middle-income countries are only above 70% and the average share is only 50%. Nearly 60% of low-income countries are below the average share of PHE to Total Health Expenditure. Overall trends of health financing indicators imply that on an average majority of LMICs have not been succeeded to attain the threshold target of PHE for the faster progress towards the UHC goals.

The health financing literature argues that the stagnant growth of PHE over the years is influenced by the unfavorable macro-fiscal policies which adversely affects the resource mobilization capacity of the economy and thereby it impedes the overall health sector development [4–6]. Experience from the richer countries argues that the favorable macro-fiscal policies^4^ (i.e. sustained economic growth, high revenue mobilization, lower fiscal deficit, and debt level) lead to higher prioritization of health expenditure but reduce the budgetary allocation during the financial crisis period [4, 8]. Earlier studies have argued that unfavorable macro-fiscal conditions have adversely affected the share of PHE in the total budget during the global financial crisis period of late 2008 in the USA [9]. The impact of the global financial crisis was felt most by the richer countries that leads to a huge reduction in budgetary allocation on health associated with increased OOP health payments due to the lower revenue generation during the post-global recession period [10, 11]. On the contrary, the insurgent of the crisis had created new fiscal policy measures – mobilization of revenue from external grants and effective utilization of health budget, as consequence there were no budgetary cuts for the health sector in Soviet Union countries [12, 13]. Similarly, several developing countries were faced with higher fiscal deficit and debt services burden during the post-global financial crisis period but there was no reduction seen in the social sector expenditure [14]. Overall literature suggests that the impact of macro-fiscal factors on the allocation of health budget may not be seen as a positive direction always and it may have an adverse impact during the period of the financial crisis. Therefore, this study has basically examined two research questions in the context of LMICs which remain unnoticed in the earlier studies –First, whether macro-fiscal policies of LMICs have had a positive influence on health financing or not; Second, whether there has been any difference in the prioritization of health budget allocation between pre- and post-global financial crisis period.

This study has examined the impact of macro-fiscal policies on health financing by adopting socio-economic factors in 85 LIMICs for the period 2000 to 2013. This study contributes to the existing literature in three important ways. First, the bulk of the literature has been investigating the impact of economic growth on PHE in LMICs, while the impact of macro-fiscal policies on PHE has remained unnoticed. Therefore, we have examined certain macro-fiscal determinants: economic growth, tax revenue, fiscal deficit, debt services payment, demographic change, and health outcome on PHE. Secondly, we have divided the LMICs into four sub-samples: pre-global financial crisis (2000–2008), post-global financial crisis (2009–2013), low-income, and middle-income. The classification of countries by level of income would examine the behavior of macro-fiscal policies towards prioritization of health expenditure during the global financial crisis period, because we assume that countries are heterogeneous in terms of revenue mobilization capacity, health status and different level of UHC targets. Thirdly, we have employed the two-step System Generalized Method of Moment (GMM)^5^ that capture all the endogeneity problem in the regression estimation by adopting appropriate instrumental variables.

## Methods

In this study, we have adopted a macro-fiscal framework to assess PHE using the government’s intertemporal budget constraint rule suggested by the Heller [7] and Tandon and Cashin [5]. The following macro-fiscal identity is written as follows:

1 $$ {G}_t+{\gamma}_t{B}_{t-1}={T}_t+{B}_t+{A}_t+{O}_t $$

Eq. (1) says *G*_*t*_ is government non-interest expenditure in time t; *γB*_*t*_ is non-discretionary debt interest payments; *T*_*t*_ is tax revenue; *B*_*t*_ is total government borrowing (i.e. domestic and foreign net of use of deposits); *A*_*t*_ is grants, and *O*_*t*_ is other sources of funds (i.e. non-tax revenue). The left-hand side of the Eq. (1) represents the expenditures of budgetary resources (aggregate expenditure), while the right-hand side of the Eq. (1) represents a generation of budgetary resources^6^ (aggregate revenue). After the generation of aggregate revenue from different sources in a financial year, the fiscal space for health of that particular year can be assessed through the proportion (*k*_*t*_) of PHE with respect to Aggregate Expenditure (AE) (See Eq. 1a). So, the percentage change in PHE with respect to AE indicates the financial commitment of government on the health sector in a particular financial year. In other words, the trends and magnitude of PHE over the years is inherently associated with the conducive macro-fiscal policies of an economy which mobilize the resources towards health sector through sustained economic growth, higher revenue mobilization, lower fiscal deficit and lower debt level [4, 5].

1a $$ {PHE}_t={k}_t\left({AE}_t\right) $$

Eq. (1a) says that if AE increases as a result of an increase in the overall components of resource mobilization (i.e. tax revenue, grants, non-tax revenue, and borrowings), then the PHE would increase by a fixed proportion (*k*_*t*_) of the government’s fiscal commitment on health sector remains unchanged. The focus from this perspective is to analyze the increment of overall resource mobilization through rising economic growth and derive the prioritization of health i.e. *PHE*_*t*_. But the prioritization of health expenditure adversely affects by a high fiscal gap (aggregate expenditure minus aggregate revenue) which lead to large debt stock and debt services payment emerge in LMICs [19]. Continuous debt service payment is reduced the government’s ability to discharge its primary responsibility for developing socio-economic infrastructure [20].

The above theoretical discussion leads us to construct the following mathematical functional form by adopting macro-fiscal determinants for the assessment of public health expenditure in LMICs:

2 $$ {PHE}_{it}=f\ \left({TR}_{it},{FB}_{it},{DEBT}_{it},{PCGDP}_{it},{AGING}_{it},{IMR}_{it}\right) $$

Eq. (2) shows that Public Health Expenditure^7^ (PHE) is the function of Tax Revenue^8^ (TR), Fiscal Balance^9^ (FB), and Debt Services^10^ (DEBT). Apart from these macro-fiscal factors, we have included other control variables such as Per Capita GDP (PCGDP),^11^ Infant Mortality Rate (IMR)^12^ and percentage of population ages 65 and above to total population (Aging).^13^ In this study, we have considered Ageing and IMR as covariates in the empirical models which exhibits the demand for health services. Earlier Literature has argued that demographic change (i.e. age structure) and health outcome (i.e. child mortality) of a country increases the demand for health services, resulting rise in the allocation of health budget [23–28]. Further, we have included the nature of tax revenue mobilization - Direct Taxes (DT) and Indirect Taxes (IT) as two of our covariates in the empirical models. The impact of the alternative tax system on PHE would exhibit the pattern of health system financing in LMICs and would able to examine the regressive components of revenue which might be the redundant impact on PHE [29–32]. As presented in the Eq. (1), external grants could be considered as one of the covariates of health financing but we have not regressed with our dependent variable (i.e. PHE) because the external health grants itself is a part of public expenditure on health.

We have included 85 LMICs on the basis of data availability of all included variables at least 3 years’ time period from 2000 to 2013. Income-wise categorization of countries is based on Global Economic Prospects Report of World Bank [33]. Additional file 1: Table S1 represents the country classification and sample selection adopted in this study. The variables adopted for empirical analysis has obtained from the World Development Indicators (WDI) online database of the World Bank [34] and Global Health Expenditure Database of the WHO [35]. The description of variables and summary statistics are shown in Additional file 3: Table S3. We have adopted PHE (as % of GDP) as our dependent variables, which predicts the extent to which the health system of a country depends on OOP expenditure. It shows that the mean of PHE (as % of GDP) is 2.91% while the tax revenue (as % of GDP) is 15.72%. We find that there a huge variation in fiscal deficit with a high standard deviation of 6.33 in LMICs. Additional file 4: Table S4 shows the result of a correlation matrix. There is a negative relationship between PHE and TR while FB shows a positive relationship to PHE.

From the simple pair-wise correlation matrix, it would not be anticipated the degree of association and magnitude of PHE with respect to the macro-fiscal factors over the years in LMICs. Therefore, we have applied a dynamic panel regression model - System GMM in the empirical estimation, which would estimate the impact of macro-fiscal dimensions on PHE for achieving UHC. By following Eq. (2), we have constructed following the log-log regression^14^ equation:

3 $$ {\displaystyle \begin{array}{c}\ln {PHE}_{it}={\alpha}_i+{\beta}_1\ln {TR}_{it}+{\beta}_2{FB}_{it}+{\beta}_3\ln {DEBT}_{it}+{\beta}_4\ln {PCGDP}_{it}\\ {}+{\beta}_5\ln {AGING}_{it}+{\beta}_6\ln {IMR}_{it}+{v}_i+{\varepsilon}_{it}\end{array}} $$

Where ln*PHE*_*it*_ is the log of PHE while the right-hand side of Eq. (3) shows the log of explanatory variables such as ln*TR*_*it*_, ln*DEBT*_*it*_ *FB*_*it*_, ln*PCGDP*_*it*_, ln*AGING*_*it*_, and ln*IMR*_*it*_. *V*_i_ is an unobserved country-specific effect; and *ε*_it_ is an unobserved white noise disturbance term; *i* = 1, …, *N* (cross-sectional units) and *t* = 2, …, *T*, (time dimensions). In Eq. (3), we have assumed that the country-specific effect *V*_i_ is constant over time and space while the slope estimates (*β*) are constrained across units. Therefore, it is called a fixed effects model. On the contrary, the random effects model assumes that *V*_i_ is not constant over time and uncorrelated with regressors. If the regressors are correlated with *v*_*i*_ and correlated with the composite error term (*v*_*i*_ + *ε*_*it*_), then random effects estimator will be inconsistent [36]. The appropriateness of using fixed effects or random effects model in the regression estimation has been verified through the Hausman test.^15^ In this study, we have first applied panel fixed effects model^16^ after the rejection in the null hypothesis of the Hausman test and it provides an efficient estimator by eliminating *v*_i_ in the Eq. (3).

In the fixed effects model, we verified that the country effects v_i_ are treated as fixed rather than random and it has correlated with the regressors. But the fixed effects model is unable to capture the possible correlation between lagged dependent variables (and possible other regressors) with the composite error term (*v*_*i*_ + *ε*_*it*_), particularly in the ‘small T, large N’ context. The resulting correlation creates a large sample bias in the estimate of the coefficient of lagged dependent variables [23]. Therefore, we have applied the following system GMM panel dynamic model^17^ as suggested by Arellano-Brover and Blundell-Bond [15, 16].

4 $$ {\displaystyle \begin{array}{c}\ln {PHE}_{it}={\alpha}_i+\gamma \ln {PHE}_{i,t-1}+{\beta}_1\ln {TR}_{it}+{\beta}_2{FB}_{it}+{\beta}_3\ln {DEBT}_{it}+{\beta}_4\ln {PCGDP}_{it}\\ {}+{\beta}_5\ln {AGING}_{it}+{\beta}_6\ln {IMR}_{it}+{v}_i+{\varepsilon}_{it}\end{array}} $$

In Eq. (4), the independent variables may contain strictly exogenous variables as well as weekly exogenous or endogenous variables (current value associated with a past value or error term). For example, x_it_ (vector of exogenous or independent variables) is said to be strictly exogenous if *E*(*x*_*it*_*ε*_*it*_) = 0 for all t and i while *E*(*x*_*it*_*ε*_*it*_) ≠ 0 for all *t* and *i* then it is treated as weakly exogenous (endogenous) variables. Further, if *x*_*it*_ shows a feedback relationship with an error term and current time period *t*, then *x*_*it*_ is an endogenous variable. In our model, we have assumed that *PHE*_*it*_ and *TR*_*it*_ are endogenous variables because current taxation capacity depends on its lagged tax collection [30, 38, 39]. On the other hand, macro-fiscal factors - FB, DEBT, and PCGDP are considered to be weakly exogenous. In Eq. (4), we have used lagged values of the explanatory variables as a gmm-style instrument for the level equation as well as an iv-style instrument for the first difference equation [37]. We have followed the two-step system GMM procedure in order to get efficient estimator that controls the panel specific autocorrelation and heteroscedasticity problems in the model [40]. We have used the Sargan test [41] for over-identifying restrictions statics for the validation of GMM dynamic models. The result of the Sargan test suggests that the regressions specifications have failed to reject the null hypothesis and our over-identified restriction in the model is valid (See Table 1).

**Table 1:** Dynamic System GMM Regression Model (Full Sample) Dep: ln PHE_it_

VARIABLES	(1)	(2)	(3)	(4)	(5)	(6)
ln PHE_it-1_	0.650***	0.655***	0.658***	0.630***	0.679***	0.674***
	(0.014)	(0.018)	(0.005)	(0.014)	(0.014)	(0.013)
ln TR_it_	0.057***				0.040***	
	(0.017)				(0.014)	
ln DT_it_		0.025***				0.023***
		(0.004)				(0.008)
ln IT_it_		−0.037***				−0.061***
		(0.005)				(0.003)
FB_it_			−0.002***		−0.002***	− 0.003***
			(3.66e-05)		(7.26e-05)	(0.0001)
ln DEBT_it_				0.0003	0.0002	0.0024
				(0.0020)	(0.0021)	(0.002)
ln PCGDP_it_	−0.003	0.007*	0.020***	0.012**	0.015*	0.001
	(0.007)	(0.004)	(0.004)	(0.005)	(0.008)	(0.005)
ln AGING_it_	0.049***	0.063***	0.104***	0.064***	0.077**	0.065*
	(0.008)	(0.018)	(0.035)	(0.012)	(0.038)	(0.037)
ln IMR_it_	−0.095***	−0.066***	−0.013	−0.034*	− 0.023	− 0.069***
	(0.020)	(0.017)	(0.019)	(0.017)	(0.027)	(0.020)
Constant	0.458***	0.462***	0.069	0.281***	0.042	0.573***
	(0.123)	(0.099)	(0.154)	(0.106)	(0.203)	(0.156)
AB test AR(2) (*p* -level)	0.233	0.214	0.541	0.222	0.551	0.609
Sargan test (*p* -level)	1.000	1.000	1.000	1.000	1.000	1.000
Mean VIF	1.69	1.66	1.67	1.76	1.57	1.58
Observations	893	893	893	893	893	893
No. of Country	85	85	85	85	85	85

Note: Sargan Chi-Square test used for over-identified restriction of the instrument in the model, AB test AR (2) to detect autocorrelation in the level series, and mean VIF for multicollinearity. Standard errors in parentheses; ***, **, * denotes the level of significance at 1, 5, and 10% respectively. ln = natural logarithm

*Source:* Author’s estimation

## Results

Using the system GMM regression approach, the study has examined the impact of macro-fiscal factors on PHE by controlling PCGDP, Aging, and IMR in LMICs. In Table 1, we have regressed PHE with macro-fiscal factors (i.e. tax revenue, direct tax, indirect tax, debt services payment, and fiscal balance) in a separate regression specification in order to examine their individual impact on PHE (see column 1–4). Further, we have regressed PHE with macro-fiscal factors in a single regression specification in order to examine their overall impact on PHE (see column 5–6).

We have found that tax revenue (TR) shows a positive and statistically significant relationship with PHE (see column 1 and 5). The result implies that at 1% incremental change in tax revenue leads to 0.057% change in PHE. We have also examined the impact of nature of tax revenue mobilization - Direct Tax (DT) and Indirect Tax (IT) on PHE in LMICs. The result shows that DT has a positive and statistically significant relationship with PHE while IT adversely affect to PHE. The result implies that at 1% incremental change in DT (i.e. revenue generated from income, profits, and capital gains) leads to 0.025% change in PHE. While, at 1% incremental change in IT (i.e. revenue generated from goods & services taxes) leads to −0.061% reduction in PHE. We find that the lagged dependent variable (ln*PHE*_*it* − 1_) show a positive and statistically significant relationship with the current allocation of PHE (ln*PHE*_*it*_). It implies that at 1% incremental change in PHE in the last year leads to less than 1% incremental change in the current year allocation of the health budget.

Table 1 shows that there is a negative and statistically significant relationship between PHE and fiscal balance (+ surplus/− deficit). It implies that at 1% increase in fiscal deficit leads to 0.003% reduction in PHE (see column 6). We find that debt services (i.e. interest payment) originated from past borrowings has shown a positive impact on PHE but it is not statistically significant (see column 4–6). It finds that per capita GDP (Per capita income) shows a positive and statically significant relationship with the growth of PHE. It implies that at a 1% increase in per capita income leads to 0.020% increase in PHE. We find that the aging population shows a positive and statistically significant effect on PHE. It implies that at 1% increase in the share of the old population (above 65) leads to a rise in PHE by 0.10% (see column 3). We find that there is a negative relationship between PHE and IMR and implies that at 1% improvement in child mortality leads to a 0.10% reduction in public expenditure on healthcare (see column 3).

In Table 1, we have done series of diagnostic tests - Sargan test for over-identifying restrictions of the instrument, Arellano-Bond (AB) test for autocorrelation, and Variance Inflating Factor (VIF) test for multicollinearity for the empirical validation of our regression models. The result shows that our regression specification is free from over-identification restrictions, no second-order auto-correlation (AR2), and no multicollinearity (see column 1–6).

### Robustness of the empirical results

In this study, we have divided the sample into four panels such as pre-global financial crisis, post-global financial crisis, low-income, and middle-income. Earlier literature argues that fiscal policy of developed countries has been affected severely by the global financial crisis and it had shown an adverse impact on the allocation of social sector spending [10–12]. The classification of total sample periods into a pre-global financial crisis (2000–2008), and post-global financial crisis (2009–2013) would show that whether the global financial crisis has influenced the changing pattern of PHE or not in LMICs. The classification of countries into the level of development (i.e. income-wise) would exhibit the revenue mobilization options adopted for health financing by LMICs and provides policy suggestions for the sustainability of PHE by adopting socio-economic factors.

Table 2 shows the impact of macro-fiscal factors on PHE during the pre-global financial crisis. We find a positive and statistically significant relationship between tax revenue mobilization and PHE by controlling PCGDP, aging, and IMR. It implies that at 1% rise in tax revenue leads to 0.106% increase in PHE. Further, we found that the direct tax shows a progressive (positive) towards health financing, while indirect tax revenue always shows a regressive (negative) on PHE in LIMI countries. The result implies that at a 1% rise in direct taxes leads to 0.027% increase in PHE while at a 1% rise in indirect taxes leads to 0.041% fall in PHE. Aging and PCGDP exhibit an expected positive sign and the result implies that at 1% rise of per capita income and old age population leads to 0.032 and 0.049% rise in PHE respectively.

**Table 2:** Dynamic System GMM Regression Model (Pre-Global Financial Crisis)

VARIABLES	(1)	(2)	(3)	(4)	(5)	(6)
ln PHE_it-1_	0.620***	0.647***	0.623***	0.603***	0.635***	0.624***
	(0.010)	(0.010)	(0.004)	(0.011)	(0.011)	(0.014)
ln TR_it_	0.106***				0.084***	
	(0.013)				(0.015)	
ln DT_it_		0.011***				0.027***
		(0.002)				(0.007)
ln IT_it_		−0.053***				−0.041***
		(0.004)				(0.008)
FB_it_			−0.001***		− 0.001***	− 0.002***
			(4.83e-05)		(6.32e-05)	(0.0002)
ln DEBT_it_				0.022***	0.017***	0.016***
				(0.002)	(0.001)	(0.001)
ln PCGDP_it_	0.009***	0.018***	0.032***	0.026***	0.010***	0.006*
	(0.0006)	(0.001)	(0.001)	(0.002)	(0.003)	(0.003)
ln AGING_it_	0.037***	0.020	0.041**	0.043***	0.049***	0.024
	(0.006)	(0.014)	(0.017)	(0.005)	(0.011)	(0.020)
ln IMR_it_	−0.038***	−0.042***	−0.001	−0.013*	−0.034***	−0.073***
	(0.009)	(0.011)	(0.008)	(0.007)	(0.011)	(0.012)
Constant	0.080	0.459***	0.063	0.137***	0.063	0.552***
	(0.055)	(0.062)	(0.066)	(0.033)	(0.055)	(0.071)
AB test AR(2) (*p* -level)	0.749	0.840	0.661	0.808	0.664	0.662
Sargan test (*p* -level)	1.000	1.000	1.000	1.000	1.000	1.000
Observations	491	491	491	491	491	491
Number of id	76	76	76	76	76	76

Note**:** Pre-Global Financial Crisis includes the sample of countries of the period of 2000–2008. Standard errors in parentheses; ***, **, * denotes the level of significance at 1, 5, and 10% respectively; ln = natural logarithm

*Source:* Author’s estimation

Table 3 shows the impact of macro-fiscal factors on PHE during the post-global financial crisis. We find that the impact of direct taxes on PHE is positive (elasticity is 0.020%) while indirect taxes shows a negative relationship with PHE (elasticity is 0.024%). We have found unexpected negative relationships between per capita GDP and PHE in the post-crisis period. This result seems to be obvious because due to lower economic growth and less revenue generation during the post-crisis period, the government usually reduce the budget allocation of developmental activities and emphasis on fiscal consolidation measures [12–14]. This argument has been reflected in our empirical estimation by observing the negative relationships between debt services payment and PHE. It implies that at 1% increase in debt services payment leads to 0.022% reduction in PHE (see column 5–6).

**Table 3:** Dynamic System GMM Regression Model (Post-Global Financial Crisis)

VARIABLES	(1)	(2)	(3)	(4)	(5)	(6)
ln PHE_it-1_	0.621***	0.575***	0.568***	0.553***	0.610***	0.610***
	(0.007)	(0.011)	(0.006)	(0.016)	(0.015)	(0.013)
ln TR_it_	−0.035				0.016	
	(0.025)				(0.012)	
ln DT_it_		0.020***				0.004
		(0.007)				(0.009)
ln IT_it_		− 0.024*				−0.069***
		(0.013)				(0.007)
FB_it_			−0.011***		−0.011***	− 0.011***
			(0.0001)		(0.0001)	(0.0002)
ln DEBT_it_				−0.008**	−0.015***	− 0.010***
				(0.003)	(0.001)	(0.002)
ln PCGDP_it_	−0.013***	− 0.016***	0.010***	−0.009***	0.005	−0.009
	(0.0007)	(0.002)	(0.003)	(0.002)	(0.006)	(0.008)
ln AGING_it_	0.065	0.267***	0.091***	0.248***	0.012	0.214***
	(0.063)	(0.032)	(0.033)	(0.048)	(0.026)	(0.038)
ln IMR_it_	−0.150***	−0.027**	−0.011	−0.010	− 0.111***	−0.044*
	(0.013)	(0.011)	(0.014)	(0.022)	(0.011)	(0.024)
Constant	0.973***	0.249***	0.229**	0.178	0.641***	0.476***
	(0.140)	(0.067)	(0.101)	(0.156)	(0.100)	(0.167)
AB test AR(2) (*p* -level)	0.103	0.086	0.086	0.086	0.104	0.089
Sargan test (*p* -level)	1.000	1.000	1.000	1.000	1.000	1.000
Observations	303	303	303	303	303	303
No. of Country	77	77	77	77	77	77

Note: Post-Global Financial Crisis includes the sample of countries of period 2009–2013. Standard errors in parentheses; ***, **, * denotes the level of significance at 1, 5, and 10% respectively; ln = natural logarithm

*Source:* Author’s estimation

Table 4 shows the result of low-income countries using panel dynamic system GMM regression model. Indirect tax revenue shows a negative impact and statistically significant relationships with PHE while revenue generated from direct tax has no impact on low-income countries. It implies that at 1% increase indirect tax revenue leads to a 0.05% reduction in PHE. Further, we find that fiscal balance shows a negative and statistically significant relationship with PHE. It implies that at 1% increase in fiscal deficit leads to a 0.003% reduction in PHE.

**Table 4:** Dynamic System GMM Regression Model (Low-Income countries)

VARIABLES	(1)	(2)	(3)	(4)	(5)	(6)
ln PHE_it-1_	0.649***	0.706***	0.658***	0.644***	0.695***	0.732***
	(0.030)	(0.027)	(0.029)	(0.030)	(0.028)	(0.026)
ln TR_it_	0.030				0.035	
	(0.028)				(0.027)	
ln DT_it_		0.017				−0.0004
		(0.018)				(0.017)
ln IT_it_		−0.041**				−0.051***
		(0.018)				(0.018)
FB_it_			−0.002***		−0.002***	− 0.003***
			(0.0009)		(0.0009)	(0.0009)
ln DEBT_it_				0.015	0.0069	0.014
				(0.010)	(0.010)	(0.010)
ln PCGDP_it_	−0.006	0.002	0.005	0.002	−0.006	− 0.002
	(0.009)	(0.010)	(0.010)	(0.009)	(0.009)	(0.009)
ln AGING_it_	−0.029	−0.009	− 0.008	−0.017	− 0.022	−0.006
	(0.028)	(0.027)	(0.028)	(0.027)	(0.027)	(0.027)
ln IMR_it_	−0.008	0.001	0.038	−0.012	−0.0007	− 0.026
	(0.031)	(0.031)	(0.030)	(0.027)	(0.027)	(0.026)
Constant	0.321	0.316	0.098	0.326**	0.222	0.492***
	(0.198)	(0.200)	(0.175)	(0.162)	(0.171)	(0.175)
AB test AR(2) (*p* -level)	0.293	0.237	0.661	0.280	0.652	0.602
Sargan test (*p* -level)	1.000	1.000	1.000	1.000	1.000	1.000
Observations	488	488	488	488	488	488
No. of Country	48	48	48	48	48	48

Note: Low-income includes the sample of both lower income and lower-middle income countries (Please see Table A1). Standard errors in parentheses; ***, **, * denotes the level of significance at 1, 5, and 10% respectively. ln = natural logarithm

*Source:* Author’s estimation

Table 5 shows the result of middle-income countries using panel dynamic system GMM regression model. We find that tax revenue shows a positive and statistically significant relationship to PHE. The result implies that at 1% incremental change in tax revenue leads to an increase in PHE by 0.14% in a year (see column 5). Further, we find a positive and statistically significant relationship between direct taxes and PHE which implies that at 1% rise of direct taxes leads to 0.025% increase in PHE annually. There is a negative and statistically significant relationship between PHE and debt services payment. The result implies that at 1% percent increase in debt payment leads to a 0.03% reduction in PHE.

**Table 5:** Dynamic System GMM Regression Model (Middle Income countries)

VARIABLES	(1)	(2)	(3)	(4)	(5)	(6)
ln PHE_it-1_	0.705***	0.722***	0.669***	0.733***	0.725***	0.746***
	(0.032)	(0.032)	(0.034)	(0.031)	(0.030)	(0.030)
ln TR_it_	0.112***				0.141***	
	(0.034)				(0.032)	
ln DT_it_		0.025*				0.017
		(0.013)				(0.013)
ln IT_it_		−0.012				0.012
		(0.018)				(0.018)
FB_it_			−0.005***		−0.005***	−0.004***
			(0.001)		(0.001)	(0.001)
ln DEBT_it_				−0.020*	−0.032***	−0.025**
				(0.010)	(0.009)	(0.010)
ln PCGDP_it_	−0.003	0.0005	0.030*	−9.37e-05	−0.025	−0.010
	(0.016)	(0.016)	(0.015)	(0.017)	(0.016)	(0.016)
ln AGING_it_	0.021	0.026	0.092**	0.043	0.049	0.036
	(0.043)	(0.042)	(0.046)	(0.044)	(0.037)	(0.040)
ln IMR_it_	−0.094***	−0.097***	−0.015	−0.067*	− 0.082***	−0.065**
	(0.035)	(0.034)	(0.035)	(0.035)	(0.030)	(0.030)
Constant	0.307	0.540**	0.022	0.482*	0.341	0.468**
	(0.250)	(0.257)	(0.265)	(0.274)	(0.231)	(0.235)
AB test AR(2) (*p* -level)	0.576	0.549	0.699	0.575	0.659	0.729
Sargan test (*p* -level)	1.000	1.000	1.000	1.000	1.000	1.000
Observations	37	37	37	37	37	37
No. of Country	405	405	405	405	405	405

Note: Middle income includes the sample of Upper middle-income countries (Please see Table A1). Standard errors in parentheses; ***, **, * denotes the level of significance at 1, 5, and 10% respectively; ln = natural logarithm

*Source:* Author’s estimation

The empirical results (Tables 1, 2, 3, 4, 5) shows the different level of elasticity of PHE with respect to macro-fiscal factors among LMICs. Tax revenue shows a positive and statistically significant relationship with PHE in full sample, pre-global financial crisis, middle-income, and coefficient value varies from 0.040 to 0.141%. Direct taxes show a positive and statistically significant relationship with PHE in full sample, pre-global financial crisis, post-global financial crisis, middle-income, and coefficient value varies from 0.011 to 0.027%. Indirect taxes shows a negative and statistically significant relationship with PHE in full sample, pre-global financial crisis, post-global financial crisis, low-income and the coefficient value varies from (−) 0.024% to (−) 0.069%. Fiscal balance (−deficit) shows a negative and statistically significant relationship with PHE in all samples and the coefficient value varies from (−) 0.001 to 0.004%. Debt service payment shows a negative and statistically significant relationship with PHE in middle-income, post-global financial crisis, and the coefficient value varies from (−) 0.008% to (−) 0.032%. On the contrary, debt services payment shows a positive and statistically relationship with PHE in pre-global financial crisis and the coefficient values vary from 0.016 to 0.022%. The result exhibits mainly two insights. First, total tax revenue and mobilization revenue through direct taxes have no impact on PHE in low-income countries; second, debt services payment and fiscal deficit have adversely affected PHE across LMICs.

## Discussion

Earlier studies have examined the determinants of PHE by adopting socio-demographic factors in the context of LMICs. On the other hand, there has very less empirical studied available that explains the impact of macro-fiscal policies (i.e. economic growth, fiscal balance, borrowings, and revenue) on the growth of PHE and how could macro-fiscal factors be inter-connected with each other in order to achieve UHC.

The recent empirical studies have examined the impact of tax revenue on PHE in LMICs in the perspective of UHC [29, 42]. They found that tax revenues are strongly positively associated with greater investment in public health, access to healthcare services and better health outcomes. Cross country studies have argued that alternative tax regimes such as income tax, taxes on goods & services have a different impact on the allocation of PHE and suggests that unproductive expenditure cuts and increase tax base are the alternative strategies to raise finance for the health sector [43, 44]. Sub-national level studies have argued that per capita tax revenue shows a positive and significant relationship with the growth of PHE in the medium to long-run [30, 45]. They suggested that conducive fiscal policies improve the revenue mobilization capacity that facilitates higher budgetary allocation towards the health sector.

Our results are similar to the past studies that examined the impact of revenue mobilization capacity (ratio of government revenue to GDP) on PHE and find a positive and statistically significant relationship between the series [26, 27, 39, 46, 47]. Similar studies have argued that there was a strong association between revenue mobilization and PHE for the faster movement towards the achievement of UHC [29, 30]. Studies also argued that most of the LMICs suffer lower revenue capacity due to unfavorable macro-fiscal policies in the short-run which takes a longer time in order to achieve the potential health outcome [30, 46]. Some studies argued that PHE and macro-fiscal policies are inter-related each other and there is the possibility of crowding out of government sources of health financing partially due to higher debt services burden [26, 38, 39, 48].

Earlier studies have found that PCGDP and domestic borrowings affect positively the PHE in the short-run [46, 49]. They have argued that the positive impact of government borrowings on PHE imply that short-term borrowings provide financial leverage to expand investment in the health sector. On the contrary, some studies found that higher borrowings reduce the current expenditure on health [38, 39, 42, 48]. So, there has a mixed response in the relationship between the level of borrowings and the allocation of PHE. Our result is similar to earlier studies that show a positive relationship between PHE and debt services payment but not statistically significant [42, 46]. On the contrary, many studies find that public debt (as a share of GDP) shows an adverse impact on PHE and they considered public debt as one of the potential sources of finance for health sector development during the period of fiscal deficit [30, 45]. Our finding corroborates to the earlier literature who find a positive relationship between PHE and PCGDP [21, 22, 28].

Our result implies that the rising fiscal deficit in a given financial year might be reduced the allocation of the health budget in long-run. Therefore, certain fiscal policy intervention in terms of alternative revenue generation would be the potential source for health financing in long-run. Our result is similar to those studies, who finds that a negative relationship between fiscal deficit and social sector spending - health and education and they imply that at 1% increase in the level of fiscal deficit leads to decrease the overall social sector spending of around 3% in a financial year [30, 42]. Many kinds of literature have proved that fiscal balance has not any direct impact on PHE rather fiscal deficit affects indirectly to the allocation of health budget via lower the level of economic growth [38, 48]. They argued that at 1% increment in budgetary surplus is associated with higher PCGDP which leads to an increase in the allocation health budget [48]. Other literature finds that fiscal rule has not any negative impact on the allocation of health budget rather fiscal deficit/surplus has always seen a positive impact on the growth of PHE [31, 33]. They argued that institutional restrictions such as a mandatory fiscal referendum, mandatory health insurance, and fiscal rule do not squeeze the share of PHE to GDP in Swiss cantons [50]. In the context of positive relationships between fiscal deficit and PHE, they argued that expenditure on health can be financed through persistently rising government borrowings from the private sector [20, 25].

Our result is similar to those studies who finds that age structure has a positive impact on healthcare expenditure [26, 27]. In the 1990s, several studies analyzed the impact of population aging on health expenditure and found ambiguous relationships between them [21, 50]. Some studies find that demographic changes explain the rising of healthcare costs and argued that a number of persons aged over 65 are a predominant factor for rising health expenditure [26, 27]. On the contrary other studies finds population aging is not associated with higher health expenditure [28, 39]. Earlier literature argues that the negative coefficient estimates between PHE and IMR could be the result of other unobserved factors, which are negatively correlated with trends in public expenditure on healthcare [28]. Another argument is that as countries experienced better health outcomes (i.e. reduce child mortality and improved longevity) in the first instance through the health expenditure leads to generate a healthier population in the long-run. Therefore, the demand for healthcare services would eventually fall which is associated with the marginal reduction in the share of health expenditure [24]. As literature suggested that a less healthy population would, on average, require more resources and thus result in greater demand for healthcare and an increase in the share of PHE [23, 50]. The result implies that government or policymaker must respond positively to the demand for healthcare services by allocating more funds to the health sector.

Our result is similar to the earlier literature who have argued that prior allocation of health expenditure is treated as an endogenous variable and determines the current allocation of health expenditure [21, 25, 38]. Some of the studies argue that the release funds for the developmental activities are delayed due to persistent rigidities in the institutional setup (i.e. budget-making process) and utilization of funds thereby current allocation of public expenditure is positively influence by previous financial year budget allocation [24, 25].

The result implies that the revenue generation through indirect taxes has reduced the allocation of health expenditure and more regressive towards health care. This can partially be happened due to less prioritization on health expenditure than the other categories of expenditure [5, 6, 45]. The result implies that both low-income and low middle-income counties need to raise revenue through improving taxes from income and wealth, and widening the tax base. Thereby the negative repercussion of fiscal deficit and debt service payment will not be a problem in the long-run. The result concludes that middle-income countries manage their fiscal deficit through tax revenue and borrowings. The overall result from the analysis of both low-income and middle-income found two insights. First, revenue mobilization towards health financing is very strong in middle-income countries. Second, low-income countries finance their health expenditure through borrowings due to lower revenue generation capacity.

There are various ways the LMICs could be mobilized domestic revenue for health financing. First, additional domestic revenues collection through non-tax revenue - natural resources such as oil, gas, and minerals; earmark revenue from taxation on tobacco and alcohol; and payroll taxes [18, 51]. Second, some innovative sources of revenue - voluntary levies on the purchase of airline tickets, mobile phone minutes, levies on the purchase of tobacco, currency transactions, value-added tax receipts from the business agreement, and secure private investment in the health system through establishing capital risk mitigation funds [52]. Third, improved tax revenue collection through different tax policy and administrative reform [53]. Fourth, political commitment in terms of imposing earmark taxes on income, profit and high prioritization of health in the national budget [54]. Fifth, the adoption of the prepayment mechanism through social insurance schemes leads to a reduction in OOP health expenditure and achieve near to UHC [55].

## Conclusions

This study has examined the impact of macro-fiscal factors - revenue mobilization, fiscal balance, debt services payment, per capita GDP, aging on PHE in LIMI countries. The study has found that tax revenue is an important source of health financing in LMICs. This study empirically verified that health financing strategy towards the achievement of UHC is not the same among the LMICS and the incremental change of health budget depends on the nature of revenue mobilization. For instance, the degree of change in PHE with respect to tax revenue is higher in middle-income countries than the low-income countries. Our study has confirmed that the impact of revenue mobilization and prioritization of health budget have not followed a systematic pattern during both pre- and post-global financial crisis period. The result shows that PHE has been affected adversely by the lower tax revenue, low level of PCGDP and higher debt services payment during the post-global financial crisis period. On the contrary, the growth of PCGDP and revenue mobilization show positive trends towards the allocation of health budget during the pre-global financial period.

This study concludes that favorable macro-fiscal policies would necessarily raise finance for the health sector development but the annual increment of health budget prioritization would be influenced by the nature of tax revenue mobilization and demand for healthcare services. Generation of health-specific revenues and effective usage of health budget probably accelerate progress towards the achievement of UHC. This study offers some interesting fiscal policy intervention for faster progress towards the achievement of UHC in any LMICS. First, efforts should make to improve tax administration by reducing tax evasion and avoidance; second, effective utilization existing financial resources and generate additional revenue through earmarked taxes would possibly enhance the fiscal resources for the health sector.

## Additional files

Additional file 1: **Table S1.** List of Sample Countries (*N* = 85, Observation = 893, T = 14 year from 2000 to 2013). (DOCX 15 kb)

Additional file 2: **Table S2.** Trends and patterns of health financing indicators (2000–2014). (DOCX 14 kb)

Additional file 3: **Table S3.** Description of Variables and Summary Statistics. (DOCX 12 kb)

Additional file 4: **Table S4.** Pair-Wise Correlation. (DOCX 15 kb)

## Data Availability

The data used for this study can be obtained from the World Development Indicators (WDI) of the World Bank, and the National Health Account (NHA) of the World Health Organization. These data are available in the public domain for research purpose.

## Abbreviations

AB: Arellano-Bond
AE: Aggregate Expenditure
AR2: Second-order Auto-Correlation
DEBT: Debt Services
DT: Direct Taxes
FB: Fiscal Balance
GDP: Gross Domestic Product
GGE: General Government Expenditure
GMM: Generalized Method of Moment
GNI: Gross National Income
IMR: Infant Mortality Rate
IT: Indirect Taxes
LMICs: Low-and Middle-Income Countries
NHA: National Health Account
OOP: Out-of-Pocket
PCGDP: Per capita GDP
PHE: Public Health Expenditure
THE: Total Health Expenditure
TR: Tax Revenue
UHC: Universal Health Coverage
VIF: Variance Inflating Factor
WDI: World Development Indicators

## Footnotes

1 Health financing focuses on how to move closer to UHC with issues related to - how and from where to raise sufficient funds for health; how to overcome financial barriers that exclude many poor from accessing health services (https://www.who.int/healthsystems/topics/financing/en/).

2 UHC embodies three related objectives - equity in access to health services, quality of health services, and financial risk protection to needy people.

3 Health financing indicators are Public Health Expenditure as percent of GDP (PHE as % of GDP), PHE as percent of General Government Expenditure (PHE as % of GGE), PHE as percent of Total Health Expenditure (PHE as % of THE), Out-of-Pocket expenditure as percent of THE (OOP as % of THE), and Per Capita PHE. Mathauer and Carrin [3] has suggested the threshold target of health financing indicators in order to provide basic healthcare packages for achieving UHC. These are PHE as % GDP (≥5), PHE as % GGE (≥15), per capita PHE (≥86$), PHE as % THE (≥70), and PHE as % OOP (≤30).

4 The Literature argues that there are no such well-defined indicators for macro-fiscal policies which drive economic growth [7]. For instance, in developing countries, the key macro-fiscal determinants include foreign direct investment, tax revenue, human capital development, demography, monetary policy, and geographic factors while in developed countries the key macro-fiscal determinants include physical capital, revenue, financial, and technological factors. But these studies have not pointed out categorically that these determinants can be used for the analysis of public health expenditure.

5 System GMM dynamic model developed by Arellano-Bover [15] and Blundell-Bond [16], and it is an advanced version of difference GMM of Arellano and Bond [17].

6 Budgetary resources can be generated by pooling revenue from different channels - first, taxes levied directly on individuals such as personal income tax and tax on corporate income or profits (direct tax); second, taxes on sales and custom duties (indirect taxes); third, revenue from government-owned enterprises or assets (e.g. revenue comes from natural resources such as oil, gas or minerals); fourth, earmarked revenues such as taxes on tobacco or alcohol and fifth, Payroll taxes. All the above taxation are the part of compulsory or prepayment, made by law and its role is predominant towards UHC [18].

7 Public health expenditure includes current and capital health expenditure from government (central and local) budgets, external borrowings and grants, and social (or compulsory) health insurance funds.

8 Tax revenue includes revenue collected from taxes on income, profits, and capital gains; taxes on goods and services; taxes on the property; and other taxes. It is compulsory transfers to the central government for the public purpose.

9 Cash surplus or deficit includes revenue (including grants) minus expenses, minus net acquisition of nonfinancial assets. It is called the overall budget balance.

10 Total debt services include some principal repayment and interest payment on both short-term and long-term borrowings.

11 PCGDP or per capita income is the proxy to measure the economic status of the population of a country and is considered to be as potential determinants for rising health expenditure [21, 22].

12 We have taken IMR as one of the covariates in the empirical model because IMR is considered as a flash indicator of the health status of the population for achieving SDGs and reduction of IMR depends on access to medicine and health facilities that intend to increase in the allocation of the health budget [23–25].

13 We have included Ageing as one of the covariates in the regression model because for two reasons [26–28]. First, the pace of population aging around the world is increasing dramatically and by 2050, 80% of all older people will live in LIMI countries. Second, the aging population is often assumed to consider a burden to society and policies should be adapted to provide financial support for health care to these people.

14 Using logarithms in the empirical models reduce the variability in the data and represent the elasticity of the dependent variable with respect to independent variables in the regression model.

15 The null hypothesis of the Hausman test says there are no systematic differences in the coefficient of fixed effects and random effects estimation. The rejection of the null hypothesis implies that we need to apply fixed effects model rather random effect model and vice versa [36].

16 The empirical results of the panel fixed effects model are not reported here and it can available upon request.

17 The System GMM dynamic model applied in certain situations such as fewer time periods (T) and many individuals (N); a linear functional relationship; dependent variable is dynamic (i.e current value is correlated of its own past value); independent variables are not strictly exogenous (i.e. correlated with past value and possibly correlated with current error term; fixed individual effects; heteroscedasticity and autocorrelation exist within individuals but not across them [37].
