# Supplementary material for: Impact of macro-fiscal determinants on health financing: empirical evidence from low-and middle-income countries
Source: Glob Health Res Policy. 2019 Aug 9;4:21. doi: 10.1186/s41256-019-0112-4 (PMC6688340; doi:10.1186/s41256-019-0112-4)
Supplement: Supplementary file 1 — Table S1. List of Sample Countries (N = 85, Observation = 893, T = 14 year from 2000 to 2013). (DOCX 15 kb) [file 41256_2019_112_MOESM1_ESM.docx]

**Table S1** List of Sample Countries (N=85, Observation = 893, T= 14 year from 2000-2013)

| **Lower Income**  **(N = 15, Obs = 149)** | **Lower-middle income**  **(N = 33, Obs = 339)** |  | **Upper-middle income**  **(N = 37, Obs = 405)** |  |
| --- | --- | --- | --- | --- |
| Afghanistan (T =7) | Armenia (T=10) | Lao PDR (T=7) | Albania (T=3) | Jordan (T=13) |
| Benin (T=13) | Bangladesh (T=11) | Lesotho (T=8) | Algeria (T=12) | Kazakhstan (T=5) |
| Burkina Faso (T=12) | Bhutan (T=10) | Moldova (T=13) | Angola (T=13) | Lebanon (T=14) |
| Cambodia (T=12) | Cabo Verde (T=5) | Morocco (T=11) | Azerbaijan (T=5) | Macedonia, FYR (T=8) |
| Central African Republic (T=9) | Congo, Rep. (T=8) | Nicaragua (T=13) | Belarus (T=13) | Malaysia (T=13) |
| Congo, Dem. Rep. (T=9) | Cote d'Ivoire (T=13) | Nigeria (T=10) | Belize (T=13) | Maldives (T=12) |
| Ethiopia (T=11) | Egypt, Arab Rep. (T=11) | Pakistan (T=14) | Bosnia and Herzegovina (T=9) | Mauritius (T=14) |
| Madagascar (T=12) | El Salvador (T=13) | Papua New Guinea (T=3) | Botswana (T=7) | Mongolia (T=13) |
| Mali (T=14) | Georgia (T=13) | Philippines (T=13) | Brazil (T=13) | Namibia (T=12) |
| Mozambique (T=3) | Ghana (T=11) | Samoa (T=3) | Bulgaria (T=13) | Paraguay (T=8) |
| Nepal (T=14) | Guatemala (T=13) | Sao Tome and Principe (T=11) | Colombia (T=6) | Peru (T=13) |
| Rwanda (T=6) | Honduras (T=11) | Senegal (T=4) | Costa Rica (T=14) | Romania (T=14) |
| Tanzania (T=4 ) | India (T=13) | Sri Lanka (T=13) | Dominica (T=13) | Serbia (T=6) |
| Togo (T=10) | Indonesia (T=11) | Syrian Arab Republic (T=8) | Dominican Republic (T=14) | South Africa (T=14) |
| Uganda (T=13) | Kenya (T=13) | Tajikistan (T=4) | Fiji (T=3) | St. Lucia (T=13) |
|  | Kyrgyz Republic (T=13) | Ukraine (T=13) | Grenada (T=13) | Suriname (T=12) |
|  |  | Zambia (T=11) | Iran, Islamic Rep. (T=10) | Thailand (T=14) |
|  |  |  | Jamaica (T=14) | Tunisia (T=13) |
|  |  |  |  | Turkey (T=6) |

**Note:** N = No of countries, T = No of years data available in each country. Income-wise categorization of countries is based on Global Economic Prospects Report of World Bank [33]. In the empirical estimation, we have combined lower income and lower middle income as the sample Low-Income countries while Upper Middle-income countries denote as the sample of Middle-income country.
