# Supplementary material for: Impact of macro-fiscal determinants on health financing: empirical evidence from low-and middle-income countries
Source: Glob Health Res Policy. 2019 Aug 9;4:21. doi: 10.1186/s41256-019-0112-4 (PMC6688340; doi:10.1186/s41256-019-0112-4)
Supplement: Supplementary file 2 — Table S2. Trends and patterns of health financing indicators (2000–2014). (DOCX 14 kb) [file 41256_2019_112_MOESM2_ESM.docx]

**Table S2** Trends and patterns of health financing indicators (2000-2014)

|  | PHE as % of GDP | | | PHE as % of GGE | | | PHE as % of THE | | | OOP as % of THE | | | Per capita PHE | | |
| --- | --- | --- | --- | --- | --- | --- | --- | --- | --- | --- | --- | --- | --- | --- | --- |
|  | ≥5% | < Mean | Mean | ≥15% | <Mean | Mean | ≥70% | <Mean | Mean | ≤30% | >Mean | Mean | ≥86$ | <Mean | Mean |
| Low |  |  |  |  |  |  |  |  |  |  |  |  |  |  |  |
| 2000 | 0 | 61.5 | 2.0 | 7.7 | 61.5 | 9.2 | 3.8 | 53.8 | 35.0 | 11.5 | 57.7 | 49.9 | 0.0 | 50.0 | 4.4 |
| 2014 | 7.7 | 65.4 | 2.6 | 11.5 | 53.8 | 10.2 | 0.0 | 46.2 | 41.7 | 23.1 | 61.5 | 38.4 | 0.0 | 53.8 | 14.8 |
| Lower Middle |  |  |  |  |  |  |  |  |  |  |  |  |  |  |  |
| 2000 | 8.3 | 56.3 | 2.6 | 6.3 | 52.1 | 9.3 | 18.8 | 56.3 | 49.4 | 20.8 | 45.8 | 45.2 | 2.1 | 66.7 | 24.0 |
| 2014 | 16.7 | 56.3 | 3.3 | 16.7 | 52.1 | 10.1 | 27.1 | 52.1 | 54.0 | 35.4 | 50.0 | 39.1 | 35.4 | 64.6 | 81.7 |
| Upper Middle |  |  |  |  |  |  |  |  |  |  |  |  |  |  |  |
| 2000 | 18.9 | 67.9 | 3.8 | 17.0 | 54.7 | 11.2 | 32.1 | 50.9 | 60.6 | 52.8 | 45.3 | 31.3 | 49.1 | 64.2 | 108.7 |
| 2014 | 26.4 | 62.3 | 4.3 | 18.9 | 56.6 | 12.1 | 32.1 | 50.9 | 63.6 | 58.5 | 45.3 | 28.2 | 100.0 | 69.8 | 333.8 |

**Note:** No. of counties included: Low = 26, Lower-Middle = 48, and Upper-Middle = 53. PHE = Public Health Expenditure, GDP = Gross Domestic Product, GGE = General Government Expenditure, THE = Total Health Expenditure, OOP = Out-of-Pocket health expenditure. ≥5 = percentage of countries PHE is more than or equal to 5% of GDP; ≥15 = percentage of countries PHE is more than or equal to 15% of GGE; ≥70 = percentage of countries PHE is more than or equal to 70% of THE; ≤30 = percentage of countries PHE is less than or equal to 30% of THE; ≥86$ = percentage of countries PHE is more than or equal to 86 US$ per population. < Mean = less than mean value (average), >Mean = more than mean value (average).

*Source:* Author’s estimation from the Global Health Expenditure Database of the WHO [35].
