# Supplementary material for: Impact of macro-fiscal determinants on health financing: empirical evidence from low-and middle-income countries
Source: Glob Health Res Policy. 2019 Aug 9;4:21. doi: 10.1186/s41256-019-0112-4 (PMC6688340; doi:10.1186/s41256-019-0112-4)
Supplement: Supplementary file 3 — Table S3. Description of Variables and Summary Statistics. (DOCX 12 kb) [file 41256_2019_112_MOESM3_ESM.docx]

**Table S3** Description of Variables and Summary Statistics

| **Variables** | **Description** | **Mean** | **Std. Dev.** | **Min** | **Max** |
| --- | --- | --- | --- | --- | --- |
| PHE | Public health expenditure (% of GDP) | 2.91 | 1.34 | 0.16 | 7.14 |
| Tax Revenue (TR) | Tax revenue (% of GDP) | 15.72 | 6.60 | 0.019 | 61.02 |
| Direct Tax (DT) | Taxes on income, profits and capital gains (% of revenue) | 21.45 | 12.09 | 0.874 | 79.53 |
| Indirect Tax (IT) | Taxes on goods and services (% of revenue) | 32.51 | 14.74 | 0.71 | 80.98 |
| Fiscal Balance (FB) | Cash surplus/deficit (% of GDP) | -1.71 | 6.33 | -19.36 | 128.11 |
| Debt Services (DEBT) | Total debt services (% of GNI) | 4.73 | 4.61 | 0.059 | 56.84 |
| PCGDP | Per capita GDP (US dollar) | 2802.11 | 2476.19 | -0.17 | 13039.12 |
| AGING | Population ages 65 and above (% total population) | 5.72 | 3.51 | 2.21 | 18.87 |
| IMR | Infant Mortality Rate (per 1,000 live births) | 36.57 | 25.94 | 3.6 | 122.6 |

**Note**: Unbalanced panel of 85 low-income and middle-income countries over the period of 2000 to 2013, and total observations 893 (See Appendix Table A1); GDP: Gross Domestic Product, GNI: Gross National Income.

*Source:* Author’s estimation from the World Development Indicators of the World Bank [35].
