# Supplementary material for: Impact of macro-fiscal determinants on health financing: empirical evidence from low-and middle-income countries
Source: Glob Health Res Policy. 2019 Aug 9;4:21. doi: 10.1186/s41256-019-0112-4 (PMC6688340; doi:10.1186/s41256-019-0112-4)
Supplement: Supplementary file 4 — Table S4. Pair-Wise Correlation. (DOCX 15 kb) [file 41256_2019_112_MOESM4_ESM.docx]

**Table S4** Pair-Wise Correlation

| Variables | PHE | TR | DT | IT | FB | DEBT | PCGDP | AGING | IMR |
| --- | --- | --- | --- | --- | --- | --- | --- | --- | --- |
| PHE | 1.000 |  |  |  |  |  |  |  |  |
| TR | 0.330*** | 1.000 |  |  |  |  |  |  |  |
| DT | -0.133*** | 0.333*** | 1.000 |  |  |  |  |  |  |
| IT | 0.142*** | 0.067 | -0.107*** | 1.000 |  |  |  |  |  |
| FB | -0.103*** | 0.110*** | -0.031 | -0.122*** | 1.000 |  |  |  |  |
| DEBT | 0.179*** | 0.261*** | 0.078 | 0.176*** | -0.032 | 1.000 |  |  |  |
| PCGDP | 0.402*** | 0.306*** | 0.160*** | 0.086 | -0.088*** | 0.250*** | 1.000 |  |  |
| AGING | 0.327*** | 0.186*** | -0.056 | 0.282*** | -0.022 | 0.209*** | 0.395*** | 1.000 |  |
| IMR | -0.422*** | -0.191*** | -0.000 | -0.286*** | 0.138*** | -0.280 | -0.599*** | -0.433*** | 1.000 |

Note: ***, **, * denotes the level of significance at 1%, 5%, and 10% respectively.

*Source:* Author’s estimation
